# Teaching an old dog new tricks: next-generation CAR T cells

**DOI:** 10.1038/s41416-018-0325-1

**Published:** 2018-11-09

**Authors:** Nicholas Tokarew, Justyna Ogonek, Stefan Endres, Michael von Bergwelt-Baildon, Sebastian Kobold

**Affiliations:** 1 Center of Integrated Protein Science Munich (CIPS-M) and Division of Clinical Pharmacology, Klinikum der Universität München, Lindwurmstrasse 2a, 80337 Munich, Germany; 2 Department of Medicine III, Klinikum der Universität München, Munich, Germany

**Keywords:** Cancer immunotherapy, Cancer therapeutic resistance

## Abstract

Adoptive T cell therapy (ACT) refers to the therapeutic use of T cells. T cells genetically engineered to express chimeric antigen receptors (CAR) constitute the most clinically advanced form of ACT approved to date for the treatment of CD19-positive leukaemias and lymphomas. CARs are synthetic receptors that are able to confer antigen-binding and activating functions on T cells with the aim of therapeutically targeting cancer cells. Several factors are essential for CAR T cell therapy to be effective, such as recruitment, activation, expansion and persistence of bioengineered T cells at the tumour site. Despite the advances made in CAR T cell therapy, however, most tumour entities still escape immune detection and elimination. A number of strategies counteracting these problems will need to be addressed in order to render T cell therapy effective in more situations than currently possible. Non-haematological tumours are also the subject of active investigation, but ACT has so far shown only marginal success rates in these cases. New approaches are needed to enhance the ability of ACT to target solid tumours without increasing toxicity, by improving recognition, infiltration, and persistence within tumours, as well as an enhanced resistance to the suppressive tumour microenvironment.

## Introduction

Immunotherapy has become an established part of the standard care for a number of different cancers, including melanoma, non-small cell lung cancer and kidney cancer.^1^ The implementation of T cell-activating strategies followed the discovery that tumour-mediated immunosuppression occurs across most tumour entities.^2^ The development of antibodies to components of inhibitory checkpoints such as programmed cell death protein 1 (PD-1) has led to unparalleled response rates and efficacy in patients who would otherwise be refractory to treatment.^3,4^ Consequently, there has been a paradigm shift in oncology with the establishment of T cells as both a therapeutic target of antibodies and an effector mechanism against the cancer cell; in this latter context, T cells are able to directly trigger apoptosis of tumour cells through granule exocytosis (perforin, granzyme) or death ligand–death receptor (Fas–FasL, TRAIL) systems.^5^

Building on this success, the direct therapeutic use of T cells, in a therapy referred to as adoptive T cell therapy (ACT), would appear as a logical progression. Three forms of ACT currently exist: first, the use of tumour-specific T cells (tumour-infiltrating lymphocytes, TILs) isolated from a patient’s resected tumour, which are expanded in vitro and therapeutically reinfused;^6^ second, the genetic engineering of T cells isolated from peripheral blood to express a T cell receptor (TCR) that recognises a specific cancer antigen;^7^ or, third, the genetic engineering of T cells using fully synthetic chimeric antigen receptors (CAR) consisting of an antigen-binding domain fused to T cell-activating moieties.^8^

The use of TILs was first reported in the late 1980s,^6^ but the difficulties in reproducibly yielding TILs across different patients and types of cancer, as well as the burden of standardisation, given the variance in quantity and quality of the starting tissue, explains the slow clinical development of this approach. By contrast, CAR T cells have been tested in a wide range of cancer types, especially haematological malignancies. CARs specific for CD19 (also known as B-lymphocyte antigen CD19) have induced high remission rates (over 80%) in patients with treatment-refractory acute lymphocytic leukaemia (ALL) or diffuse large B-cell lymphoma.^9,10^ Although it still remains to be proven, it is very likely that these remission rates will also prolong overall survival for these otherwise untreatable patient populations. These findings have prompted the FDA to approve anti-CD19 CAR T cells as the first T cell therapy even for refractory ALL and diffuse large B-cell lymphoma.^11,12^ More CARs are currently undergoing clinical development for the treatment of other haematological entities, such as chronic lymphocytic leukaemia, or low grade lymphomas and myeloma.^13,14^ It is consequently likely that there will be an increase in approvals for the use of CAR T cells across a wider array of malignancies.

However, although the efficacy of CAR T cell therapy is unchallenged for a number of haematological malignancies, it is important to realise that, with over 100 types of cancer, haematological cancers comprise only a small fraction of diagnosed cancers and are responsible for only 6% of all reported deaths.^15^ Attempts over the past couple of years to treat solid malignancies with CAR T cells have resulted in elevated toxicities and a minimal observable therapeutic benefit for patients,^16,17^ highlighting the heterogeneity inherent in the therapeutic response of different cancer types. Here, we review the evolution of the CAR T cell over time, with a particular focus on the current limitations of CAR T cell therapy in solid tumours and strategies to overcome these limitations.

## Evolution of CAR T cell development

**Fig. 1:**
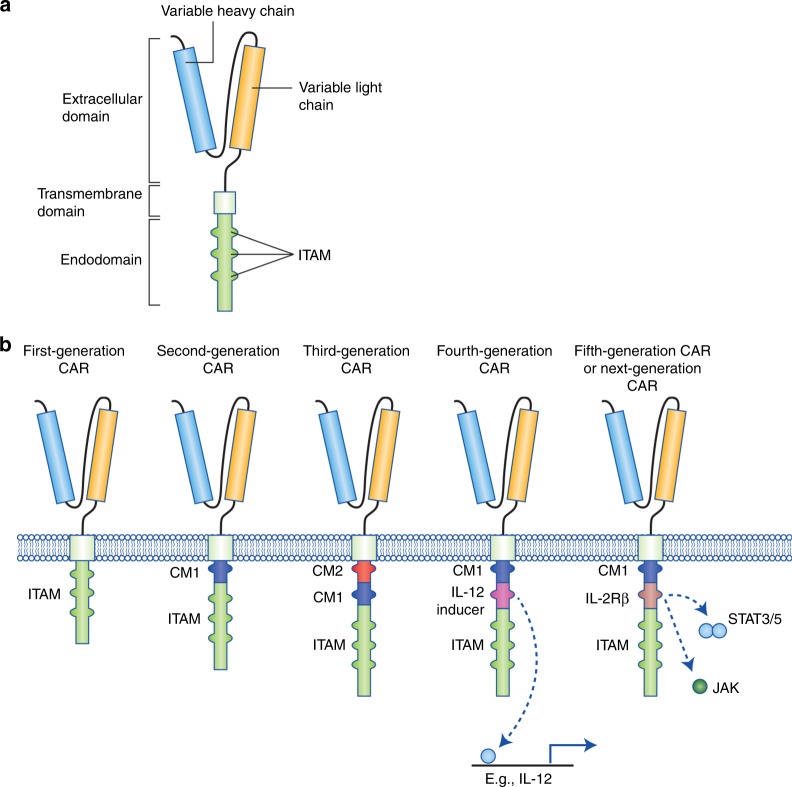
Structure of different chimeric antigen receptor (CAR) generations. **a** The core structure of a CAR, highlighting the major components of the extracellular domain, the transmembrane domain and the intracellular domain (endodomain). **b** Evolution of the development of CARs from the first generation, which contained only ITAM motifs in the intracellular domain. Second-generation CARs included one co-stimulatory molecule (CM)1, and third-generation CARs contained a second CM. The fourth generation of CARs was based on second-generation CARs (containing 1–3 ITAMs) paired with a constitutively or inducibly expressed chemokine (e.g. IL-12). These T cells are also referred to as T cell redirected for universal cytokine-mediated killing (TRUCKs). The fifth, or ‘next generation’, is also based on the second generation of CARs, with the addition of intracellular domains of cytokine receptors (e.g. IL-2Rβ chain fragment). ITAM immunoreceptor tyrosine-based activation motifs, CD co-stimulatory domain, IL-12 activation of interleukin 12 transcription, IL-2Rβ truncated intracellular interleukin 2β chain receptor with a STAT3/5 binding motif

CARs are bioengineered receptors with specificity directed towards a desired antigen. The first CARs were generated some 30 years ago and have subsequently undergone a stepwise evolution in their development.^1,18^ The versatility of CARs stems from the fact that, unlike innate TCRs, they can recognise antigens in the absence of presentation by the major histocompatibility complex (MHC).^18^ This is a particular advantage when MHC expression is lost as a result of the immunosuppressive network of cancer cells.^19^ CARs comprise three main components: the extracellular domain, which is responsible for antigen recognition, the transmembrane domain, and the intracellular signalling domain.^20^ The extracellular region can be further segmented into the signal peptide, which is cleaved from the mature CAR expressed at the cell surface,^21^ and the antigen-recognition domain. The antigen-recognition domain is a single-chain fragment variant (scFV) predominantly composed of the variable light and heavy chain regions of an antigen-specific immunoglobulin separated by a flexible linker, and is tethered to the transmembrane domain through the spacer, which transmits the receptor-binding signal.^20^ The transmembrane domain is usually a hydrophobic alpha helix that spans the cell membrane and is fundamental for surface expression and stability of the receptor.^20,22^ The third region is the intracellular domain (or endodomain). Following antigen binding, the intracellular domain clusters and undergoes conformational changes, which enables downstream signalling proteins to be recruited and phosphorylated.^23,24^ The endodomain can contain several functional units. The intracellular domain of the T cell co-receptor CD3ζ, which contains three immunoreceptor tyrosine-based activation motifs (ITAMs) that are important for signal transduction, is the core component of most CARs^20^ (Fig. 1a).

The progress in the development of CARs over the past three decades can be roughly grouped into five CAR generations based on the structure and composition of the endodomain.^20^ The first generation of CARs contained a single CD3ζ intracellular domain. Initial experiments with first-generation CAR T cells showed low cytotoxicity and proliferation owing to the lack of co-stimulatory (e.g. CD27, CD28, CD134, CD137) and cytokine (e.g. interleukin (IL)-2) signalling.^20,25^ A second generation of CARs was generated to enhance T cell proliferation and cytotoxicity by adding a co-stimulatory domain such as sections of CD28 or CD137 to the intracellular signalling domain.^26–28^ The third generation of CARs further expanded on the second generation by adding a third intracellular signalling sequence of an additional co-stimulatory domain such as CD134 or CD137.^20^ The fourth generation of CARs is based on second-generation CARs, but includes a protein, such as interleukin 12 (IL-12) that is constitutively or inducibly expressed upon CAR activation. T cells transduced with these fourth-generation CARs are referred to as T cells redirected for universal cytokine-mediated killing (TRUCKs). Activation of these CARs promotes the production and secretion of the desired cytokine to promote tumour killing though several synergistic mechanisms such as exocytosis (perforin, granzyme) or death ligand–death receptor (Fas–FasL, TRAIL) systems.^5,25^ TRUCKs will be further discussed in a separate section below. A fifth generation of CARs is currently being explored; these are based on the second generation of CARs, but they contain a truncated cytoplasmic IL-2 receptor β-chain domain with a binding site for the transcription factor STAT3. The antigen-specific activation of this receptor simultaneously triggers TCR (through the CD3ζ domains), co-stimulatory (CD28 domain) and cytokine (JAK–STAT3/5) signalling,^29^ which effectively provides all three synergistic signals (further discussed below) required physiologically to drive full T cell activation and proliferation. Additional variants of the aforementioned CARs, such as dual CARs, split CARs and inducible-split CARs, have been generated to further enhance the specificity and control of the transfused T cells. These CARs will be discussed in greater detail below^18^ (Fig. 1b).

In the past three decades, CARs have progressed from their initial characterisation to FDA approval for use in patients. In spite of these advances, however, it is important to bear in mind that the novel CAR designs and improvements in recent generations as seen in in vitro or in animal models have not been further corroborated in patients. In other words, no study has yet compared first or subsequent generation CARs specific for a single antigen in a clinical study, which prevents a proper comparison across the different generations of CARs and obscures the selection of optimal combinations for future clinical trials. Thus, any consideration of the use of a given CAR generation is based on preclinical animal models and not clinical data. Currently it is unclear which design would provide the best clinical benefit for patient outcome.

## Limitations to CAR T cell therapy in solid tumours

A recent clinical trial treating patients suffering from acute B-cell lymphoblastic leukaemia with CD19-specific CAR T cells have shown an unprecedented clinical outcome, with 81% of 75 treated patients going into complete remission and minimal residual disease negativity.^11,30^ By contrast, several attempts to treat solid cancers with first-generation CAR T cells directed against several antigens (carbonic anhydrase IX (CAIX), CD171, folate receptor alpha (FR-α), GD2, human epidermal growth factor receptor 2 (HER2), mesothelin, EGFRvIII or vascular endothelial growth factor receptor 2 (VEGF-R2))^31^ did not significantly benefit patients, showing limited activity and frequent toxicity.^32–34^ Using TCR-modified T cells, clinical trials have also reported severe, even lethal toxicities.^35^ A TCR targeting the cancer testis antigen NY-ESO-1 showed promising results across trials and entities, and objective responses were observed in melanoma, sarcoma and myeloma, but otherwise responses have been anecdotal.^36^

In any case, targeting solid tumours with either approach presents a number of challenges not encountered when targeting blood malignancies. The hypoxic, poorly vascularised and extracellular matrix-rich tumour microenvironment prevents T cells from infiltrating the tumour tissue. Even for such T cells that do reach the tumour, the loss of tumour antigens prevents their specific recognition. Furthermore, inhibitory surface proteins, cytokines or soluble products of disrupted cell metabolism within the tumour can impair the activation and persistence of T cells. The need to enhance T cell recruitment into solid malignancies is highlighted by the observation that elevated lymphocyte infiltration is a powerful positive prognostic marker in various cancer subtypes (e.g. breast cancer, colorectal cancer, ovarian cancer, non-small cell lung cancer, melanoma and others).^37,38^ Accordingly, there is an extensive body of literature which is focused on transforming immune-scarce ‘cold’ tumours into immune-abundant ‘hot’ tumours,^39^ a subject which will be discussed in greater detail below. A detailed collection of overall strategies outside of ACT has been recently reviewed.^40,41^

**Fig. 2:**
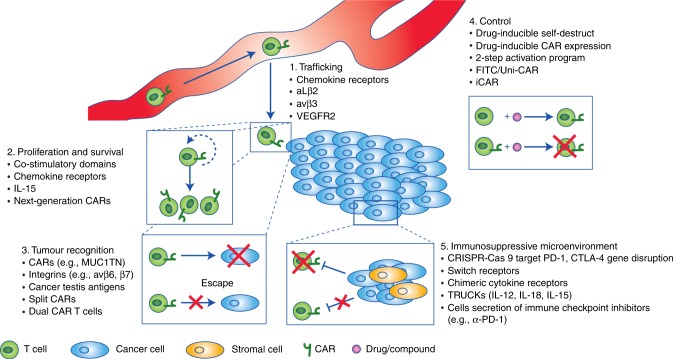
Strategies to address limitations to CAR T cell therapy in solid tumours. Overview of the five major challenges facing adoptive T cell therapy in treating solid tumours—trafficking, proliferation and survival, tumour cell recognition/discrimination, control of transfused T cells and circumventing the immune-suppressive tumour microenvironment—and some approaches currently under investigation to improve them. IL interleukin, VEGFR2 vascular endothelial growth factor receptor 2, Uni-CAR Universal CAR, iCAR inhibitory CAR, TRUCKs T cell redirected for universal cytokine-mediated killing

The importance of increased tumour recognition by T cells is underscored by several adaptive techniques adopted by cancer cells to circumvent immune detection, such as the aforementioned downregulation of MHC-associated antigen presentation.^19^ This is also further exemplified by encouraging results of phase II clinical trials of some cancer vaccines such as Canvaxin and GVAX, which were used to treat melanoma and prostate cancer, respectively.^42,43^ The mechanism of action of cancer vaccines is thought to drive antigen cross presentation and diversity, enabling the establishment of a protective immunity against cancer.^44^ Although the phase II clinical trials of Canvaxin and GVAX showed encouraging results, the phase III clinical trials of both cancer vaccines failed to demonstrate a survival benefit. Along the same lines, a multipeptide vaccine showed promising activity in phase I/II trials, paralleled by the induction of immune responses,^45^ but the phase III trial failed to achieve its primary endpoint, presumably due to low immunogenicity.^45^ These exemplary results indicate the correlation between the induction of a specific immune response and benefit to vaccine therapy, but at the same time question the overall therapeutic benefit of this approach to treat solid malignancies.^46,47^ In addition, the need to enhance T cell activation and persistence has been illustrated by the unprecedented success rate of trials of therapies that target immune inhibitory checkpoint proteins such as PD-1.^3,4,48^ Finally, many of the aforementioned notions are related to the ability of a cancer to directly alter its microenvironment, driving immune cell exclusion and reducing antigen presentation and lymphocyte activation.^49^ Based on these experiences and on preclinical studies, five important concepts have been identified which will need to be addressed to employ engineered T cells as a viable therapy for solid tumours (Fig. 2): improving T cell recruitment to tumours; enhancing T cell survival and activation; increasing tumour cell antigen recognition; implementing control strategies; and counteracting the immunosuppressive microenvironment.

## Improvement of T cell trafficking to solid tumours

Immune infiltrate is typically low or absent in tumour biopsy samples from patients who do not respond to immune checkpoint blockade therapy,^18^ and is a prognostic factor for poor overall survival.^50^ For immune therapy to be effective, effector T cells need to reach their target cells. Unsurprisingly, if CAR T cells cannot access their target cells, it is very unlikely that they will be effective at controlling tumour growth. A recent strategy to promote immune cell recruitment to tumours takes advantage of cancer chemokine signalling.^50^ Chemokines are cytokines with chemotactic abilities, and are involved in regulating migration and trafficking of various immune cells and some somatic cells.^50^ Several studies have shown that tumour cells can produce chemokines, either themselves or through their stroma, which signal back to the cancer cells to promote cancer cell proliferation, survival, progression and migration.^50,51^ Some of these chemokines can promote the recruitment of immune-suppressive cells, which further enhances the immune-suppressive tumour environment.^50^ Approaches by us and others have sought to utilise the tumour chemokine signalling network to drive T cell recruitment by engineering the expression of a cognate chemokine receptor—CCR2,^52^ CCR2b,^53,54^ CCR4,^55,56^ CCR7,^57^ CXCR2^58^ or CXCR4^59^—on the surface of CAR T cells (Fig. 1). Recently, CXCR3 has also gained considerable attention in enhancing ACT.^60^ This interest stems from the observation that PD-1 blockade and/or chemotherapy has been shown to promote ACT recruitment in a CXCR3-ligand-dependent manner.^61,62^ A growing body of evidence corroborates the observation that the chemokine landscape within a given tumour can be extremely heterogenous,^63^ illustrating the need to identify specific candidates and strategies to enhance T cell infiltration into different cancers, which will vary from patient to patient.

While transgenic chemokine receptors come with the promise of directed recruitment to a desired site, the non-tumour specificity of chemokines might ‘distract’ T cells from their intended tumour target.^64^ Undesired effects might thus range from reduced activity through to novel toxicities, which is a particular risk when the primary target of the T cell is not tumour-specific. T cell entry into non-inflamed or non-tumour tissues is low under steady-state conditions.^65^ One might hypothesise that true added toxicities might especially arise when this state is disrupted, in the case of injury or autoimmune disorders.^66,67^ Such patients will need to be excluded from the first clinical trials dealing with chemokine-receptor-transduced T cells. Along the same lines, T cells will naturally utilise endogenously expressed chemokine receptors to traffic, and this process might compete with the newly introduced receptor.^68^ Although not yet seen in studies published so far, a careful analysis of the homing behaviour of such cells will need to be considered. From the safety perspective, the consequences of aberrant chemokine receptor signalling in T cells will need to be considered to prevent unwanted side effects stemming from this axis. On the other hand, the attraction of CAR T cells to distinct chemokine gradients might promote the development of chemokine loss or downregulation as a novel escape mechanism and thereby alter cancer biology, eventually rendering subsequent lines of immunotherapeutic treatment less likely to impact on the patient’s condition. Heterogeneity of chemokine expression will also need to be clarified both within entities and patients, as some disease sites with no or lower chemokine expression might remain inaccessible to the modified T cells. Currently, novel strategies to induce tumours to express a desired chemokine ligand through several intratumoural delivery methods (further discussed below) are being explored to circumvent this issue.

A recent approach to further enhance CAR T cell infiltration into solid tumours exploits the process of T cell egress: for example, by using α4 integrin mutant (S988A), protein kinase A (PKA)-mediated phosphorylation can be inhibited, stabilising the α4 (S988A)–paxillin interaction and resulting in an increase in α4 integrin signalling. The inhibition of PKA-mediated α4 integrin phosphorylation enhances integrin αLβ2 (LFA-1)-mediated migration, a phenomenon termed integrin transregulation.^69^ Together, increased α4 and αLβ2 integrin signalling promotes T cell extravasation from the vasculature and into the tissue, promoting T cell adhesion to the vasculature of inflamed tissue in an ICAM-1- and VCAM-1-dependent manner. In in vitro experiments, the inhibition of α4 integrin phosphorylation promoted αLβ2-mediated T cell migration, while in vivo, the α4 (S988A) mutant mice showed a marked increase in T cell entry into ectopically transplanted melanoma tumours and reduced the growth of implanted B16 melanoma tumours.^70^ CAR T cells have also been engineered to target antigens such as α_v_β3 integrin, which are selectively enriched in the tumour microenvironment,^71^ or vascular endothelial growth factor (VEGF) receptor-2 (VEGFR2), a highly expressed antigen enriched in the tumour vasculature.^72^ Limited T cell infiltration into solid tumours will need to be addressed in order to drive CAR T cells into tumours that are not readily accessible or are hidden in dense extracellular matrices.

Alternatively, a technologically less challenging and clinically feasible strategy to overcome the issue of CAR T cell migration and tumour infiltration is to employ local chemokine delivery or production: several trials have investigated intracompartmental or intratumoural delivery of adenovirus chemokine expressing virus (CCL17)^73^ or DNA plasmids encoding the desired chemokine ligand (CCL5).^74–76^ Although these approaches are clinically feasible and do not influence the safety profile of CAR T cells, the results obtained from the trials were modest. Local delivery also comes with the major caveat that most patients are not candidates for such a treatment due to the position and technical difficulty of reaching the tumour site; furthermore, metastatic sites will generally remain unaffected by this approach.

## T cell recognition and targeting of tumour cells

The ability to transduce ex vivo T cells with antigen-specific TCRs or CARs provides ACT with the powerful ability to selectively target desired antigen(s). Theoretically, this ability to select and target an antigen, or antigens, should provide the transfused T cells with an elevated level of antigen discrimination and, consequently, safety. At the same time, antigen recognition is the prerequisite for any efficacy expected from T cell therapy. However, several obstacles hinder proper targeting of antigen-specific CAR T cells. First, for T cell-mediated therapy to be effective, the targeted tumour antigen must be expressed on the cell surface, but most known tumour specific antigens are intracellular molecules or mutated versions of them.^77,78^ Second, the targeted antigen needs to be ideally expressed only on tumour cells—for example, truncated or mutated proteins. This is in contrast to tumour-associated antigens (TAA), which have an enriched expression in tumour cells but are also expressed in healthy tissues.^79,80^ A separate category comprises antigens expressed only in embryonic or otherwise immune privileged tissues, such as cancer testis antigens.^81^ A comprehensive overview of antigens currently targeted by CAR T cells has been recently published.^16^

In the absence of antigens that are truly tumour-specific, most T cell therapies in clinical trials are facing toxicity issues due to ‘on target off tumour’ recognition of the targeted antigen on normal cells.^18^ Several approaches are under investigation to mitigate the effects of on target off tumour recognition (discussed below). On the other hand, the major reason for treatment failure of the most advanced CAR T cells targeting CD19 is loss of antigen expression or selection by B-cell acute lymphoblastic leukaemia cells for mutants or variants that cannot be recognised by the anti-CD19 CAR T cells.^82^ Accordingly, highly specific antigen targeting by CAR T cells is accompanied by an inherent dilemma that applies selective pressure for the development of variants which are responsible for the relapse. Significant work has focused on reducing on target off tumour cross reaction by identifying more specific cancer antigens, such as αvβ6 or MUC1TN, which are highly expressed on several cancer subtypes (colon, lung, breast, cervix, pancreas and others) and can arise from post-translational cancer-specific modifications such as glycol modifications.^83,84^ In other cases, novel epitopes are exposed upon conformational changes induced by integrin activation, as seen for integrin β7, which is a candidate for targeting in multiple myeloma^85^ (Fig. 2). Currently, both on target off tumour and off target cross reaction are very difficult to predict in vitro and in silico, as shown by several examples in which preclinical models did not adequately predict effects or side effects.^86,87^ Along these lines, novel CARs need to be carefully assessed and clinicians need to favour a minimal anticipated biological effect level (MABEL) approach over the no observed adverse effect levels (NOAEL) for T cell dosing. This is of particular importance as the absence of evidence of toxicity does not equate to evidence of absence.

**Fig. 3:**
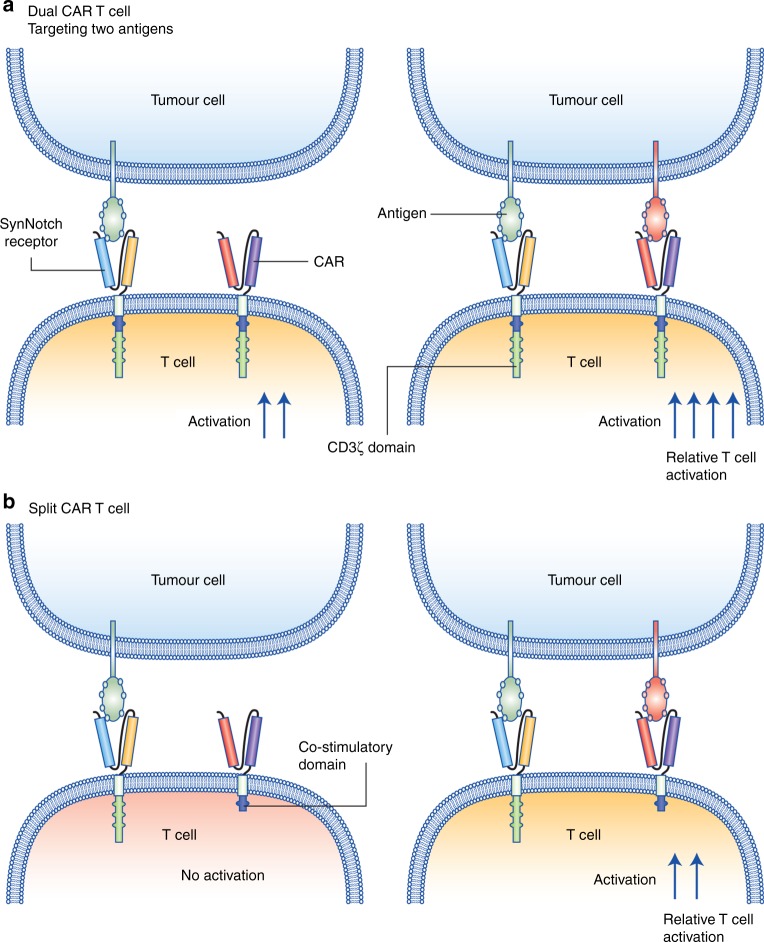
Schematic representation of selected strategies to enhance CAR T cells recognition and activation. Recent strategies to enhance the activation of CAR T cells include the use of dual CARs, targeting two surface antigens (**a**) and split CARs (**b**). Legend indicated in the figure

With the lack of bona fide cancer-specific antigens, efficacy and safety might be further enhanced by simultaneously targeting two or more different cancer-associated antigens. The advantage of this approach is that fully fledged T cell activation would only occur when both antigens are present, which should ideally be a very rare event outside of a tumour. With this in mind, two avenues of investigation are being pursued. The first combines the expression of two identical—except for the targeted antigen—CARs (referred to as dual CARs),^88^ enabling an enhancement of efficacy when both antigens are engaged (Fig. 3a). The second involves separation of the co-stimulatory domains (e.g. CD28 and 41BB) from CD3ζ on two different CARs (referred to as split CARs),^89^ requiring the simultaneous engagement of both CARs to complete T cell activation (Fig. 3b). Both approaches have demonstrated strong efficacy in preclinical models.^90–92^ Their clinical relevance is currently being investigated in clinical trials (including NCT03289455, NCT03125577, NCT03258047, NCT03198052). Additional strategies to enhance antigen discrimination and to reduce on target off tumour killing have also been engineered and are further explored in the section on programmed T cell control below.

## T cell proliferation and survival

A major predictor of the efficacy of CAR T cell therapy is the extent of T cell expansion and persistence in a given patient.^93^ Expansion and persistence need to occur in order for T cells to reach a sufficient number to effectively eliminate the target tumour cells. Fundamental immunology indicates that T cells require three synergistic signals to drive proliferation and survival: TCR engagement, co-stimulatory signalling and cytokine signalling.^94^ The solid tumour microenvironment, however, despite containing plenty of antigens, lacks activating molecules and instead exposes effector cells to an abundance of suppressive molecules. This environment drives T cell anergy and dysfunction.^94^ A strategy to promote CAR T cell function involves the addition of co-stimulatory signalling moieties such as CD28 or 41BB to the CAR itself to promote T cell expansion and survival in these non-permissive environments.^18^ Mechanistically, however, the addition of one or more co-stimulatory domains might not be sufficient when promitotic signals are rare, prompting additional investigations into alternative mechanisms which can further enhance T cell proliferation and maintenance. One strategy is to fortify the T cells with cytokine signalling in such a T cell hostile environment (Fig. 2). Such an approach using repetitive dosing with biologically active IL-15 in mice enhanced antigen-specific CAR T cell recruitment, infiltration, proliferation and cytotoxic capabilities.^95^ This concept of ‘fortification’ is further realised in fifth-generation CARs, which include a truncated IL-2 receptor β chain and a STAT3-binding moiety. Activation of this kind of CAR can drive comprehensive TCR signalling complete with co-stimulatory and cytokine-driven JAK–STAT signalling to enhance proliferation and survival of the bioengineered T cells.^29^ These approaches to promote T cell proliferation and survival are still in the early stages of investigation and their potential contribution to future clinical settings is uncertain.

## Programmed T cell control mechanisms

**Fig. 4:**
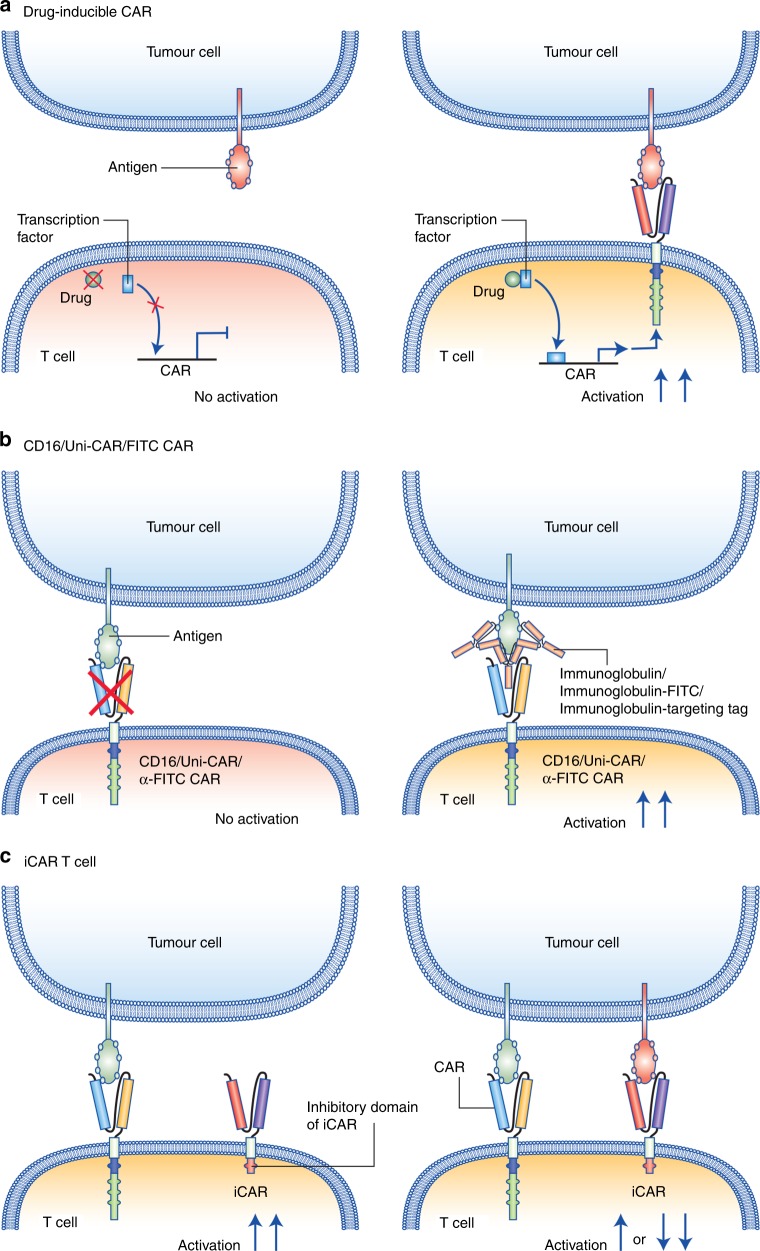
Schematic representation of selected strategies to inhibit CAR T cell activation and prevent off tumour on target activity. Strategies to enhance CAR T cell safety include: **a** drug-inducible CAR T cells, **b** CAR T cells that require antigen-specific antibodies (CD16/FITC or Uni-CAR T cells) and **c** inhibitory CARs (iCARs), which moderate or inhibit T cell activation when antigens expressed on bystander cells are encountered. Legend indicated in the figure

As highlighted above, compromised safety and CAR-related side effects are major hurdles to CAR T cell application.^18,86,87^ Once transfused into patients, CAR T cells are biologically active and difficult to control. With several CAR-associated deaths in clinical trials, there is a critical need to enhance and control CAR safety. In principle, safety could be enhanced in three ways: conditional and controllable activation of CAR T cells through switch compounds, such as a modified version of rapamycin (referred to as ‘rapalog’)^96^ (Fig. 4a); depletion of CAR T cells upon the occurrence of undesired and uncontrolled side effects; and suppression of CAR activity in the vicinity of non-tumour cells through receptors that recognise the latter. The most advanced modalities are strategies to deplete T cells upon the occurrence of side effects, using a suicide gene or agents to deplete cells bearing specific markers through monoclonal antibodies. An example of the latter is CAR T cells that express a truncated human EGFR polypeptide (huEGFRt), which is devoid of the extracellular N-terminal ligand-binding domains and intracellular receptor tyrosine kinase domain but can be targeted by cetuximab. Infusion of cetuximab, which is an IgG1 antibody, will bind to the truncated EGFR expressed on the transfused T cells and be eliminated by other immune cells through the recognition of the constant domain of cetuximab in a fragment crystallisable (Fc) receptor-dependent manner.^97,98^ Other examples include ganciclovir-mediated targeting of T cells transduced with the herpes simplex tyrosine kinase^99^ or a chemical inducer of dimerisation to deplete T cells transduced with an inducible caspase 9.^100^ Although in the context of allogeneic stem cell transplantation, some evidence suggests T cell depletion to be sufficient to reverse graft-versus-host-disease, such a relationship has not yet been proven for CAR T cell side effects.^97^ It remains to be seen whether depletion of CAR T cells upon the occurrence of severe toxicities could revert or prevent lethal or long-lasting tissue damage. An important side effect of such an approach, however, is the removal of the biologically active CAR T cells, which could in effect promote cancer recurrences.

Another attractive option is to render CAR T cell function dependent on the provision of additional substances such as antibodies or derivatives thereof. Along these lines, CARs targeting the Fc part of antibodies (e.g. anti-CD16-CAR),^101^ or targeting tags appended to antibodies (Uni-CAR)^102^ or derivatives such as FITC (anti-FITC CAR),^103^ have been developed (Fig. 4b). A similar strategy is the use of bispecific antibodies that specifically recruit CAR T cells by targeting a co-transduced marker antigen decorating the CAR T cell for enhanced CAR activity.^104^ These approaches allow precise control of CAR T cell reactivity based on the antibodies’ half-life, thus lowering the risk of side effects while preserving efficacy. Another advantage of aforementioned therapies is the possibility of using a single cellular product, irrespective of the TAA to be targeted, in combination with an approved monoclonal antibody.

Lastly, CAR T cells can utilise a NOT-gate circuits system to enhance efficacy and reduce off target targeting (Fig. 4c). A NOT-gate circuit is a CAR T cell that expresses (either inducibly or constitutively) two or more different CARs. The first CAR would target a tumour-specific antigen and contain the mandatory stimulatory (CD3ζ) and co-stimulatory (e.g. CD28 or CD137) domains, while the second CAR would be specific to an antigen which is typically expressed on normal healthy tissue or bystander cells and linked to inhibitory (iCAR) signalling domains (e.g. PD-1 and CTLA-4). The simultaneous engagement of both the CAR and the iCAR within the same immune synapse would prevent or dampen the activation of the T cell, and result in poor activation or T cell anergy,^42^ thus enhancing discrimination between tumour cells and healthy cells. These novel strategies to improve the specificity and control of CAR T cells could potentially increase the discrimination of normal versus tumoural cells, but additional investigation into the clinical significance is needed.

## Counteracting the immunosuppressive microenvironment

Solid tumours are typically composed of a heterogeneous population of cells in a microenvironment which is hypoxic, poorly vascularised, has an elevated interstitial pressure and is often surrounded by a dense extracellular matrix.^51^ These physical and metabolic barriers prevent immune cell recruitment, activation and persistence, while simultaneously promoting the recruitment of immune suppressor cells.^49,51^ This enables tumour cells to escape immune detection and destruction and to dampen CAR T cell activity.^105^ Accordingly, immune suppression needs to be overcome for optimal CAR T cell action. In principle, three main avenues have been explored to enable CAR T cell activity in spite of immune suppression: deletion of selected immune-suppressive factors in the therapeutic T cells; introduction of molecules able to circumvent T cell suppression in CAR T cells; and combination therapies providing exogenous immune suppression reversion (Fig. 2).

Novel approaches to circumvent tumour-driven immune suppression have been explored by using the clustered regularly interspaced short palindromic repeats (CRISPR)–CRISPR-associated 9 (Cas9) system. CRISPR–Cas9 technology has been used to enhance CAR T cell function by knocking out genes encoding T cell inhibitory receptors or signalling molecules such as PD-1 or CTLA-4.^106–108^ PD-1-deficient CD19 CAR T cells show improved killing of a CD19-positive and programmed cell death ligand 1 (PD-L1)-positive cell lines in vitro and enhanced clearance of tumour xenografts in vivo.^106^ Similarly, disruption of PD-1 enhanced in vivo anti-tumour activity in a model of prostate cancer using CAR T cells specific for prostate stem cell antigen (PSCA).^108^ Interestingly, the feasibility of generating PD-1 and CTLA-4 dual inhibitory pathway-resistant PSCA-specific CAR T cells has been demonstrated.^107^ However, the deletion of inhibitory molecules can be a double-edged sword, as the removal of some suppressive pathways may lead to uncontrolled proliferation or to an increased risk of autoimmunity. Immune checkpoints are molecules normally involved in the maintenance of peripheral tolerance to self-molecules by preventing over-reactivity of self-antigen-specific T cells. Deletion of such important molecules might induce, aside from overactivation, off-target activation of T cells, as observed previously in studies with systemic PD-1 antibody blockade.^109^ Although the use of CRISPR-Cas9 technology to knock out genes in CAR T cells is still in preclinical studies, a clinical trial that will evaluate the effect of PD-1 knockout by this technology in NY-ESO-1-specific TCR-transduced T cells has recently been approved (NCT03399448).^110^

An alternative approach to prolong the responsiveness of CAR-modified T cells is to equip them with activating chimeric switch receptors (CSRs), also referred to as immunomodulatory fusion proteins. Activating CSRs combine the extracellular ligand-binding domain of an inhibitory receptor (PD-1 or CTLA-4) fused through a transmembrane domain with the cytoplasmic co-stimulatory signalling domain of CD28.^111–115^ The engagement of the extracellular portion of this fusion receptor transmits an activating signal instead of the normal physiological inhibitory signal. T cells transduced with PD-1–CD28 CSR have been shown to have increased extracellular signal-regulated kinase (ERK) phosphorylation and granzyme B expression, increased proliferation and cytokine secretion—indicative of T cell co-stimulation—upon PD-L1 binding.^112^ The potent anti-tumour activity of CTLA-4–CD28 CSR T cells has been further validated in a murine model of melanoma.^113^ Two phase I clinical trials have already been initiated to determine the safety and efficacy of autologous PD-1–CD28 CSR-modified T cells in the treatment of PD-L1-positive, recurrent and metastatic malignant tumours (NCT02930967) and recurrent glioblastoma (NCT02937844). In line with the encouraging results from studies combining TCR-modified T cells and CSR,^111,113,114^ PD-1–CD28 can improve the therapeutic effects of anti-mesothelin CARs and anti-PSCA CARs in tumour xenograft models.^115^ The improved therapeutic effect was attributed to an increase in the number of infiltrating CAR T cells, a decrease in susceptibility of the cells to tumour-induced hypofunction, and an attenuation of inhibitory receptor expression. Together, these results provide a foundation for further clinical studies using CAR-T cell therapy in combination with a CSR strategy.

A further embodiment of CSRs is provided by chimeric cytokine receptors, which can transform secreted immunosuppressive signals into immune-activating signals. This notion has been demonstrated for the chimeric cytokine receptor IL-4R–IL-7R, which comprises the anti-inflammatory IL-4 receptor (IL-4R) exodomain fused to the pro-inflammatory IL-7 receptor (IL-7R) endodomain.^116^ Upon receptor engagement by tumour-derived IL-4, immunosuppressive effects were abrogated, increasing proliferation and activation of tumour-directed cytotoxic T cells and enhancing the anti-tumour activity in vivo. Optimal activation of tumour-antigen-specific T cells overexpressing IL-4R–IL-7R CSRs occurred only when engineered T cells encountered their specific tumour antigen and elevated IL-4 was present in the tumour microenvironment. In such a system, IL-4 would mimic beneficial IL-7 signalling through the CSR. Most cytokine signals mainly act in conjunction with TCR or related signalling, which is provided in this system by the CAR. Along these lines, full activation will only be seen in the presence of both IL-4 and the CAR target.^117^ These results encourage further adaptation of CSRs as a supplementary method to fine-tune CAR T cell therapies. It remains unclear whether targeting one of such inhibitory pathways would be sufficient to achieve therapeutic efficacy in a clinical setting.

Another approach involves the use of truncated suppressive receptors such as a dominant-negative form of TGF-β receptor II or a dominant-negative PD-1, which shield T cells from the negative effects of TGF-β and PD-L1, respectively.^118–120^ Apart from the upregulation of inhibitory ligands such as PD-L1, the tumour microenvironment is additionally enriched with immunosuppressive cytokines, one of which is TGF-β. TGF-β is produced in excess by tumour cells themselves, as well as cells of the tumour stroma.^121^ TGF-β has been shown to impair both innate and adaptive cellular immunity, generating a favourable microenvironment for tumour growth and metastasis, and is deemed an essential component of the tumour’s anti-immune defences.^122,123^ Following this line of thought, strategies that combine CAR T cell therapy with blocking immune-suppressive axes such as PD-1–PD-L1 or providing important cytokines depleted in the tumour environment, have been investigated. Combining PD-1-blocking antibodies with CAR T cell therapy has dramatically enhanced CAR T cell function in different preclinical models and prompted the initiation of clinical trials in different indications.^124,125^ Cytokine support, the third signal required for T cell activation, can also be a means of breaking through T cell suppression: provision of IL-2 or IL-15 has yielded significant improvements in different models,^126,127^ although a major caveat is the side effects of exogenous cytokine application. It remains to be seen if these deleterious effects might be outweighed by the clinical benefits of CAR T cell therapy.

## CAR T cells as factories

A more complex strategy for overcoming tumour-driven immune suppression involves the use of CAR T cells that are capable of transforming the immunosuppressive microenvironment into an immune-permissive one. This strategy involves the use of the fourth generation of CARs, or TRUCKs, which are CAR T cells engineered to constitutively or inducibly express pro-inflammatory cytokines. One such candidate is IL-12, which strongly enhances the response of innate and adoptive immune cells to cancer cells.^128^ IL-12 increases interferon (IFN)-γ secretion and the expression of granzyme B and perforin by T cells^129^ and NK cells,^130^ and suppresses tumour-induced T-regulatory (T-reg) cell proliferation.^131^ As a consequence, these mechanisms may enhance additional tumour clearance by bystander NK cells and conventional T cells, and counteract the ability of T-reg cells to promote tumour growth. These effects will naturally synergise with CAR T cells for enhanced anti-tumoural activity. For this reason, the combination of CAR T cell therapy with constitutive or inducible IL-12 expression has been extensively explored for the treatment of several malignancies in preclinical models.^132–134^ IL-12-expressing TRUCKs exhibited a remarkable efficacy against solid tumours in preclinical models, with no observable signs of toxicity, as compared to clinical trials studying the use of recombinant human IL-12 as a therapy.^135^ Furthermore, a clinically relevant advantage of IL-12-producing TRUCKs is the elimination of established cancer without the requirement for cyclophosphamide preconditioning.^132^ The first dose-escalation trials of autologous TILs transduced with a gene encoding IL-12 driven by a T cell nuclear factor showed anti-tumour activity in melanoma, but also displayed dose-limiting severe toxicities including liver dysfunction, high fevers, and sporadic life-threatening haemodynamic instability.^136^ A phase I clinical trial of CAR T cells targeting the Mucin 1 antigen and co-expressing IL-12 provided only little therapeutic benefit, although no adverse side effects were reported.^137^ More clinical trials examining the safety and efficacy of IL-12-armoured CAR T cells are currently ongoing.^138^

A safer alternative to TRUCK IL-12 T cells might be CAR T cells secreting IL-18. IL-18 is a cytokine characterised as an inducer of IFN-γ expression in T cells^139^ and has been shown to activate monocytes and lymphocytes without causing severe toxicity in clinical trials.^140^ Recently, two studies revealed that inducible expression of IL-18 in CAR T cells enhances proliferation and anti-tumour activity of monocytes and lymphocytes.^141,142^ Interestingly, IL-18-producing TRUCK T cells induced acute inflammatory reactions and altered the balance of pro-inflammatory and anti-inflammatory cells in established, large pancreatic and lung tumours. Specifically, IL-18 polarises CAR T cells towards an effector phenotype paired with an acute inflammatory response. IL-18 CAR T cell treatment was accompanied by an increase in the number of M1 macrophages and NK cells, whereas a decrease in M2 macrophages, T regs and suppressive dendritic cells was observed. These effects go beyond the CAR T cell-only effects, and synergise with their activity.^142^

Another cytokine that has been overexpressed in CAR T cells is IL-15. IL-15 is a regulator of T cell homoeostasis, prolonging T cell survival. Additionally, it increases the lytic capacity of T cells by stimulating granzyme B expression.^143^ Anti-CD19 CAR T cells engineered to secrete IL-15 showed improved antigen-driven expansion, reduced PD-1 expression and cell death, and improved anti-leukaemic efficacy.^144^ In another approach, anti-CD19 CAR T cells have been designed to secrete a soluble form of herpes virus entry mediator (*HVEM*, *TNFRSF14*).^145^ The *HVEM* gene is frequently mutated in germinal centre (GC) lymphomas.^146^ The loss of inhibitory cell–cell interactions between *HVEM* and B and T Lymphocyte Attenuator (BTLA) leads to autonomous activation of B-cell proliferation and drives the development of GC lymphomas in vivo.^145^ *HVEM*-deficient lymphoma B cells also induce a tumour-supportive microenvironment. Accordingly, *HVEM* protein secreted by modified CAR T cells binds BTLA and restores tumour suppression.

Another strategy that exploits CAR T cells as local delivery agents or ‘micro-pharmacies’ is combinatorial immunotherapy, in which engineered CAR T cells secrete immune checkpoint inhibitors. For example, CAR T cells engineered to secrete human anti-PD-L1 antibodies to block T cell exhaustion have been shown to clear renal cell carcinoma in a humanised mouse model.^147^ Anti-PD-L1 antibody delivery to the tumour site led to a five-fold reduction in tumour growth and a 50–80% reduction in tumour weight in comparison to treatment with parental CAR T cells. Moreover, expression of PD-L1 and the cell proliferation marker Ki67 in the tumours decreased and levels of secreted granzyme B by modified CAR T cells increased. Anti-CD19 CAR T cells engineered to secrete anti-PD1 antibody enhanced anti-tumour activity and prolonged overall survival in a xenograft mouse model.^148^ Interestingly, a comparison of combinatorial therapy using CAR T cells engineered to secrete anti-PD1 antibodies versus CAR T cell therapy administered in conjunction with anti-PD1 antibodies revealed that systemically injected anti-PD-1 antibody had little effect on CD8+ T cell function.^148^ This result suggests that, given the low concentration of secreted anti-PD-1 in comparison to systemic injection (15-fold lower than the amount detected in the group in which antibodies were systemically injected,^148^) the anti-PD-1 antibody secreted by CAR T cells might provide a safer and more potent approach to enhancing the functional capacity of CAR T cells.

Taken together, the delivery of different payloads to the tumour through CAR T cells has shown promise in preclinical studies. Several clinical trials have been initiated to test the safety and efficacy of CAR T cells that, in addition to targeting a specific tumour antigen, secrete either anti-PD-1 alone or anti-PD-1 in combination with anti-CTLA-4 or anti-PD-L1 antibodies (NCT03179007, NCT03182816, NCT03182803, NCT03030001, NCT02873390, NCT02862028, NCT03170141). Further development of these combination therapies may become possible by new strategies to engineer T cells.

## Conclusions

CAR T cells designed to express CD19 have shown unprecedented clinical success in otherwise refractory patients suffering from ALL or diffuse large B-cell lymphoma, frequently accompanied by severe adverse toxicity. These results exemplify the power of the approach and have revolutionised the concept of future blood-borne cancer treatments. By contrast, little or no clinical efficacy has so far been reported using CAR T cells for solid malignancies. Based on published clinical and preclinical trials with CAR-modified T cells, we have identified five important limitations to CAR therapy that need to be overcome for optimal treatment efficacy and safety: T cell recruitment, activation and proliferation, tumour cell targeting, control mechanisms, and circumventing the immune-suppressive microenvironment. These limitations will all have to be tackled in some way in order to increase T cell efficacy in solid tumours and to broaden the applicability of the strategy. An important approach will be the combination of several layers of engineering in one cellular product to address these limitations. This is an as yet unresolved issue, as most of the advances so far have been made in the area of tumour targeting, or on individually addressing these limitations as separate entities. Ongoing and future trials will reveal if the promise of cellular and, more specifically, CAR T cell therapy will benefit a broader population of tumour patients than those suffering from rare refractory haematological malignancies.
